# Comparison of nucleic acid testing and enzyme-linked immunosorbent assay in screening Hepatitis-B Virus in blood samples

**DOI:** 10.12669/pjms.42.4.13203

**Published:** 2026-04

**Authors:** Hanxiao Xin, Wei Luo, Pengyun Zhao

**Affiliations:** 1 Hanxiao Xin, Department of Blood Transfusion, Baoding NO.1 Central Hospital, Baoding, Hebei 071000, China; 2 Wei Luo, Department of Blood Transfusion, Baoding NO.1 Central Hospital, Baoding, Hebei 071000, China; 3 Pengyun Zhao, Department of Blood Transfusion, Baoding NO.1 Central Hospital, Baoding, Hebei 071000, China

**Keywords:** Enzyme-Linked Immunosorbent Assay, Hepatitis-B Virus Screening, Nucleic Acid Testing, Voluntary Blood Donation

## Abstract

**Objective:**

To investigate the value of nucleic acid testing (NAT) and enzyme-linked immunosorbent assay (ELISA) in the screening of Hepatitis-B virus (HBV) in blood samples from voluntary blood donors.

**Methodology:**

This was a retrospective observational study. Five hundred blood samples from healthy blood donors collected by the Baoding No.1 Central Hospital between from January, 2024 to January, 2025 were selected as the study subjects, and each sample was divided into two groups, one for HBV NAT and the other for ELISA. Afterward, based on the diagnostic results of the gold label test paper, the diagnostic efficacy of two detection methods for HBV was compared using sensitivity, specificity, and accuracy, compared their respective window periods for HBV using t-test.

**Results:**

The sensitivity, specificity, and accuracy of HBV NAT were higher than those of ELISA, and the window phase for NAT of the virus was shorter than that of ELISA (p < 0.05). The HBV-DNA levels were significantly different between patients with HBsAg (+)/HBeAg (+)/Anti-HBc (+), HBsAg(+)/Anti-HBe(+)/Anti-HBc (+), and infection (p=0.00), showing a positive correlation with disease severity (r =0.73, p = <0.001).

**Conclusion:**

NAT may significantly improve the specificity, sensitivity, and accuracy of HBV detection in blood samples from voluntary blood donors, with a shorter window phase than ELISA, which can better ensure blood safety.

## INTRODUCTION

Hepatitis-B virus (HBV) is the main cause of Hepatitis-B and other viral diseases, which is mainly transmitted through blood and is harmful. As a retrovirus and DNA virus, HBV can be transmitted through blood and blood products, posing a significant threat to blood transfusion safety. Its clinical manifestations include loss of appetite, nausea, upper abdominal discomfort, liver pain, fatigue, and significantly decreased liver function. If not effectively controlled, this disease may lead to various liver diseases such as cirrhosis.^1^ As of now, there are about over 250 million HBV-infected individuals worldwide, making rapid diagnosis and treatment of this disease a global public health issue.^2^ HBV infection is a sensitive social issue and can restrict individuals in their education, daily life, and work, especially in certain special positions and industries.

HBV is mainly transmitted through blood, with clinical blood transfusion being the most common method of transmission.^3^ Therefore, blood donation regulations clearly stipulate that HBsAg-positive blood should not be used for clinical blood transfusion. There is a challenge of a window phase in HBV testing. In order to reduce the risk of virus infection through blood transfusion in blood recipients and ensure the safety of blood for clinical use, it is of great clinical significance to accurately diagnose whether blood donors are infected with HBV. Hepatitis-B surface antigen (HBsAg) is a diagnostic marker for HBV infection, the accurate determination of which can facilitate rapid diagnosis of Hepatitis-B.^4^

In view of this, HBV screening for blood samples is of utmost importance. Enzyme-linked immunosorbent assay (ELISA) is the most commonly used and typical qualitative method for Hepatitis-B, which can quickly and simply determine the presence of Hepatitis-B virus surface antigen (HBsAg), antibodies, etc., at a relatively low cost, making it suitable for large-scale and high-volume physical examination screenings. However, some scholars^5^ have suggested that this detection method has poor sensitivity and may miss the diagnosis of blood samples with low concentrations of viruses. In recent years, NAT has been used clinically to detect HBV. This molecular biological detection method can directly determine the HBV-DNA levels and then respond to the presence of Hepatitis-B infection, with a high diagnostic sensitivity.^6^ Therefore, this study selected blood samples from 500 voluntary blood donors for comparative research, observing the application value of nucleic acid testing and enzyme-linked immunosorbent assay in screening for hepatitis B virus, and providing guidance for the detection of blood samples from voluntary blood donors.

## METHODOLOGY

This was a retrospective study. Five hundred blood samples (male=270, female=230) from healthy blood donors collected by the Baoding No.1 Central Hospital between from January 2024 to January 2025 were selected as the study subjects, and blood donors were 18 to 47 years old, with an average of 33.68±12.75 years, each sample was divided into two groups, one for HBV NAT and the other for ELISA. Afterward, the diagnostic efficacy of the two detection methods for HBV was calculated and compared based on the diagnostic results of the gold-label test paper method, along with a comparison of their respective window phases for HBV.

### Ethical approval:

The study was approved by the Institutional Ethics Committee of Baoding No.1 Central Hospital (No.:2022047; Dated: November 04, 2022), and written informed consent was obtained from all participants.

### Inclusion Criteria:

- Blood donors aged 18 to 50 years.
- All are voluntary unpaid blood donors.
- Specimens were tested for HBsAg by gold-labeled test strip and deemed qualified after determination of alanine aminotransferase (ALT) levels.

### Exclusion Criteria:

- Donors with failed blood specimens;
- Donors who failed to meet the relevant requirements of health examinations.^7^

Blood samples from all subjects were qualified after being tested for HBsAg by the gold-labeled test strip and ALT levels by dry chemical method. Afterward, the samples were divided into two tubes, coded and labeled with collection dates, centrifuged at a speed of 3,000 r/min for 20 min in a centrifuge, and the qualified blood samples after centrifugation were stored in a sample storage cabinet at 2°C - 8°C for future use.

All tested blood samples met the requirements and underwent initial testing and retesting. After blood sample collection, each sample was divided into two 5 mL sample tubes: one contained EDTA-K2 anticoagulant vacuum gel, and the other tube contained coagulant vacuum gel. NAT and ELISA were performed on each sample, respectively, and labeled with a unique barcode. Afterward, the blood samples were centrifuged and stored in a thermostat at 2°C-8°C, and all blood samples were required to be tested within 48 hours after collection.^8^ ELISA: The STAR enzyme immunoassay analysis sampling system and the automated FAME24/20 enzyme immunoassay analyzer were used, with negative and positive control groups, quality control groups, and blank control groups established according to the instructions. The testing process was completed following standard operating procedures. Reagents, test samples, and HBsAg quality control products were equilibrated for at least 30 minutes within the laboratory’s required temperature and humidity range.

The concentrated washing solution was shaken and then diluted using ultrapure water as required by the instructions. Sample diluent, negative control, positive control, samples, and quality control were loaded using the automated STAR enzyme immunoassay analysis sampling system. After the loading of samples, a manual visual inspection was conducted to check for any omissions, followed by a double check. In case of omissions, the corresponding samples were manually supplemented. Additionally, the automated enzyme immunoassay analyzer was used to perform the testing according to the established testing procedure. Throughout the experiment, the testing personnel closely monitored the operation of FAME, especially during the steps of plate loading, reagent addition, and plate washing.

Meanwhile, the loading/unloading of the enzyme plate should be handled gently to prevent overturning while correctly loading the enzyme plate, thus ensuring that the barcode scanned by the computer accurately matches the barcode on the enzyme plate at the corresponding position on the plate rack. Observed results: Negative control A value ≤ 0.1; Positive control A value ≥ 0.8; QC ≥ Cutoff value. If the internal quality control violates the -3s rule, the experiment is considered invalid.^9^ NAT method: A real-time fluorescence quantitative PCR system, nucleic acid extractor, nucleic acid amplification instrument, and corresponding reagents were used to complete the detection of HBV DNA according to the instrument’s SOP and reagent instructions. Ig(HBV-DNA) > 2.7 was deemed positive.

### Outcome Measures:

1) The diagnostic efficacy of ELISA and NAT was compared and analyzed according to the results of the gold-labeled test strip, with sensitivity, specificity, and accuracy as efficacy indicators; 2) The differences in window phase between the two detection methods were compared and analyzed; 3) The HBV-DNA levels and HBV-DNA positive proportion under different infection status were compared and analyzed.

### Statistical Methods:

SPSS 20.0 software was used for the statistical analysis of all data, and measurement data were expressed as (*x̅*+*s*). According to the Shapiro-Wilk test, all data conforms to a normal distribution, inter group data analysis was performed using the t-test, categoriocal data were expressed via n (%),and rates were compared by the χ^2^-test, multi group comparison using one-way ANOVA and correlations expressed using Pearson correlation coefficients. P< 0.05 was considered statistically significant.

## RESULTS

In 500 blood donors, 50 were positive and 450 were negative for HBV (HbsAg) by gold-labeled test strip, among which 38 were positive and 462 were negative by ELISA, while 38 were positive and 462 were negative by NAT. The sensitivity, specificity, and accuracy of the latter were significantly higher than those of the former, and the difference was statistically significant (p<0.05) (Table-I and II).

**Table-I:** Correlation Analysis between ELISA, NAT, and Gold-Labeled Test Strip.

Gold-labeled test strip	ELISA			NAT		
	Positive	Negative	Total	Positive	Negative	Total
Positive	25	25	50	36	14	50
Negative	13	437	450	2	448	450
Total	38	462	500	38	462	500

**Table-II:** Comparative Analysis of Diagnostic Efficacy of 2 Detection Methods (*x̅*+*s*).

Group	Sensitivity*	Specificity*	Accuracy*
ELISA	50% (25/50)	97% (437/450)	92% (462/500)
NAT	72% (36/50)	99% (448/450)	97% (484/500)
*χ* ^2^	5.09	8.20	9.47
*p* *	0.02	0.004	0.002

***Notes:***

* *χ*^2^ test

The window phase was 22.64±5.38 days and 28.87±5.71 days for NAT and ELISA, respectively, with the former seeing a shorter window phase than the latter, and the difference was statistically significant (p<0.001) (Table-III).

**Table-III:** Analysis of Differences in Window Phase between the 2 Detection Methods (*x̅*+*s*).

Indicator	Window Phase (d)*
NAT	22.64±5.38
ELISA	28.87±5.71
t	4.89
p^△^	<0.001

***Notes:***

△ independent-sample t-test

Among the positive samples by gold-labeled test strip, 23 cases were with HBsAg(+)/HBeAg(+)/Anti-HBc(+), 16 were with HBsAg(+)/Anti-HBe(+)/Anti-HBc(+), and 11 were with viral infection. NAT and comparative observation of HBV-DNA levels in the 3 types of samples indicated some differences. Compared with patients with infection, HBV-DNA levels were significantly different in those with HBsAg(+)/HBeAg(+)/Anti-HBc(+) and HBsAg(+)/Anti-HBe(+)/Anti-HBc(+) (P<0.001), with a positive correlation with the severity (r = 0.73, P<0.001) (Table-IV).

**Table-IV:** Comparison and correlation Analysis of HBV-DNA Detection under different infection status (r) (*x̅*+*s*).

Group	n	HBV-DNA Levels*	Correlation*
Infectious Stage	11	4.20±1.46	
HBsAg positive	16	5.46±1.73	
HBsAb positive	23	8.37±1.62	
F/r		29.49	0.73
p^△^		<0.001^△^	<0.001^※^

***Notes:***

△ one-way ANOVA;

※ Pearson Correlation Coefficient.

## DISCUSSION

This study confirmed that the sensitivity, specificity, and accuracy of HBV NAT are higher than those of ELISA (p=0.00), indicating that NAT has a higher value in determining the presence of HBV infection in blood samples from blood donors. Additionally, the analysis suggests that in the early stages of HBV infection, patients may not exhibit any clinical symptoms. By comparison, ELISA primarily evaluates diseases based on the degree of coloration after the enzyme complex binds with antibodies, while information on antigens and antibodies has not yet formed during the window phase or virus variation, which can interfere with the probability of ELISA. Specifically, NAT is a quantitative detection method that can directly detect early viral DNA infection through DNA amplification technique and determine its level, thus providing a higher diagnostic value. The criteria for determining the “window phase” infection are HBV DNA positivity, with all five makers for Hepatitis-B as negative.^10^ This study demonstrates that the window phase for NAT and ELISA is 22.64±5.38 days and 28.87±5.71 days, respectively, with the former seeing a shorter window period than the latter, and the differences are statistically significant(p=0.00). The main reason for this is the covert nature of HBV infection. ELISA primarily detects diseases by using enzyme complexes and antibody binding for coloration, making it susceptible to factors such as the virus infection window phase and virus variation, which may lead to misdiagnosis. In contrast, NAT can detect viral nucleic acid that appears earlier in blood samples through the nucleic acid amplification technique. In recent years, with the continuous development of medical technology, NAT has gradually been widely used in blood screening. As a novel molecular biological detection method, NAT involves extracting samples such as blood and respiratory secretions from patients to detect the presence of foreign viral nucleic acid, thereby determining the presence of viral infection. In HBV screening, NAT can be used to determine the levels of HBV DNA, so as to identify whether the patient’s blood sample is infected with the virus.^11^ Meanwhile, the clinical use of NAT has the characteristics of a high specimen recovery rate and ease of automation, making it a supplementary method for serological clinical diagnosis, suitable for the detection and screening of a large number of blood samples. Studies have revealed^12^ that the NAT of the virus utilizes molecular biology detection methods, employs PCR technology for viral nucleic acid amplification, and sequences the amplified DNA, thereby determining the presence of HBV and the viral DNA content. Since this method can objectively and accurately reflect the level of HBV replication in vivo, an increasing number of blood stations are using NAT as part of infectious disease screening of blood samples from blood donors.

Additionally, quantification of NAT can more directly and effectively reflect the actual situation of HBV infection in the body, leading to results with high sensitivity and specificity. Furthermore, it has been shown^13^ that HBV-DNA levels in blood samples from individuals with HBsAg(+)/HBeAg(+)/Anti-HBc(+) are significantly higher than those in HBsAg(+)/Anti-HBe(+)/Anti-HBc(+) blood samples and infection samples. The findings of this study also confirmed that there are significant differences in HBV-DNA levels among patients with HBsAg(+)/HBeAg(+)/Anti-HBc(+), HBsAg(+)/Anti-HBe(+)/Anti-HBc(+), and infection period (P=0.00), and these levels are positively correlated with severity (r=4.66, p=0.00). Therefore, HBV-DNA levels can largely reflect the degree of HBV transmission. Moreover, as HBV-DNA levels in the body of subjects increase, the virus becomes more infectious. Clinically, the infectivity of HBV can be determined through the quantitative determination of HBV-DNA levels.

The main sources of HBV transmission include both acute and chronic HBV patients as well as virus carriers, and HBV is infectious in the blood whether at the onset or incubation period.^14^ Blood transmission is the primary route of HBV transmission, with blood transfusion and blood products being the most common ones.^15^ The severity of liver cell damage and HBV immunopathological damage varies due to different intensities of the host’s immune response to HBV infection. In developing countries, there is a high prevalence of HBV infection, with a high rate of HBV-positive detection among blood donors during blood screening.^16^ Therefore, strengthening the blood screening of voluntary blood donors to prevent the transfusion of HBV-contaminated blood samples can effectively prevent HBV transmission through blood transfusion, thus effectively preventing HBV transmission.^17^ After initial infection and extensive replication of the virus, the human immune system gradually recognizes the virus, presents antigen information, and activates various immune cells. Laboratory tests can initially detect HBsAg positivity, and as the virus continues to replicate and the immune mechanism is fully established, Hepatitis-B virus core antigen (HBcAg), Hepatitis-B virus e antigen (HBeAg), and corresponding antibodies can also exhibit positivity. The HBV window phase refers to the period in which viral infection has occurred but antibody information has not yet formed.

Specifically, the individual has already been infected with the virus during the window phase, but the viral antibody detection suggests negative due to incomplete or unstable formation of antibodies against HBV,^18^ resulting in missed diagnosis. As one of the most important reasons for scrapping blood during blood quality screening, the HBV window phase not only causes waste of blood from blood donors but also reduces the safety margin of blood for clinical use. Therefore, it is of great significance to select the best screening method for HBV detection, distinguish the blood of voluntary blood donors in a timely manner, and promptly exclude the blood of individuals with HBV infection for the prevention and control of HBV disease. Since HBV is a blood-borne virus, blood testing for blood donors is the optimal approach to preventing and controlling HBV infection. In clinical diagnosis, ELISA is widely used due to its low cost and convenience,^19^ making it significant in blood examination. Specifically, ELISA is a traditional method for HBV diagnosis and can maintain excellent immunoreactivity, with the main detection indicators of antigens and antibodies. However, Alzubiery et al.^20^ indicated that this method has lower sensitivity for HBV detection and cannot quantitatively estimate the virus content in samples. On the one hand, it cannot completely detect the samples with low virus content concentration.

On the other hand, it lacks viral numerical records, making it difficult to objectively present the current virus content and the degree of disease transmission risk, which is not conducive to evaluating treatment efficacy in clinical applications. With the development of medical laboratory techniques, the third-generation ELISA technique can not only perform qualitative detection of HBV but also quantitative evaluation, despite being a semi-quantitative detection, and it relies on the results of the S/CO ratio to determine the presence and severity of infection, with the higher values indicating higher levels of HBsAg, HBsAb, etc. However, Bahrami et al.^21^ suggested that the S/CO ratio-based detection efficacy of the third-generation ELISA is only slightly higher than that of the traditional ELISA. These findings show some drawbacks in relying solely on ELISA for blood sample screening of blood donors, which poses a significant safety hazard for subsequent clinical use of blood. Additionally, due to the relatively long window phase of ELISA,^22^ some positive blood samples with infection risk are not easy to detect using this method, which may lead to misdiagnosis.

### Limitations:

However, this study comes with the limitations of a small sample size. Additionally, the combination of detection methods for a certain virus in clinical practice is significantly superior to a single detection method.

## CONCLUSION

Both NAT and ELISA exhibit high detection rates in the detection of HBV in blood samples from blood donors. However, in terms of sensitivity, specificity, misdiagnosis rate, and window phase, NAT demonstrates more significant advantages, which may serve as an auxiliary method for detecting HBV in blood donors in combination with ELISA, thereby providing more reliable clinical screening results and improving transfusion safety.

### Recommendations:

In the future, the sample size should be increased, along with the combined application of different detection methods for HBV, so as to realize more accurate detection of HBV in blood donors and provide greater safety and protection for blood recipients.

### Authors’ Contributions:

**HX:** Designed this study and prepared this manuscript, and are responsible and accountable for the accuracy or integrity of the work.

**WL:** Collected and analyzed clinical data. Critical Review.

**PZ:** Significantly revised this manuscript.

All authors have read and approved the final manuscript.
